# Cardiac Modulation by *Santolina chamaecyparissus* Aqueous Extract in a Rat Model of Mammary Carcinogenesis

**DOI:** 10.3390/cimb48060599

**Published:** 2026-06-05

**Authors:** Tiago Azevedo, Jessica Silva, Maria J. Pires, Mário Ginja, Tiane C. Finimundy, Maria J. Neuparth, Manuela Matos, Lillian Barros, Paula A. Oliveira, Ana I. Faustino-Rocha

**Affiliations:** 1 Centre for the Research and Technology of Agro-Environmental and Biological Sciences (CITAB), Institute for Innovation, Capacity Building and Sustainability of Agri-Food Production (Inov4Agro), University of Trás-os-Montes and Alto Douro, 5000-801 Vila Real, Portugal; silva_jessy@hotmail.com (J.S.); joaomp@utad.pt (M.J.P.); mginja@utad.pt (M.G.); mmatos@utad.pt (M.M.); pamo@utad.pt (P.A.O.); 2 Animal and Veterinary Research Centre (CECAV), Associate Laboratory for Animal and Veterinary Sciences (AL4AnimalS), University of Trás-os-Montes and Alto Douro, 5000-801 Vila Real, Portugal; 3 CIMO, LA SusTEC, Instituto Politécnico de Bragança, Campus de Santa Apolónia, 5300-253 Bragança, Portugal; tiane@ipb.pt (T.C.F.); lillian@ipb.pt (L.B.); 4 Research Center in Physical Activity, Health and Leisure (CIAFEL), Faculty of Sports—University of Porto (FADEUP), 4200-450 Porto, Portugal; mneuparth@hotmail.com; 5 Laboratory for Integrative and Translational Research in Population Health (ITR), 4200-450 Porto, Portugal; 6 Toxicology Research Unit (TOXRUN), University Institute of Health Sciences, CESPU, 4585-116 Gandra, Portugal; 7 Comprehensive Health Research Center (CHRC), Department of Zootechnics, School of Sciences and Technology, University of Évora, 7004-516 Évora, Portugal

**Keywords:** breast cancer, cardiotoxicity, echocardiography, hemodynamic function, phenolic compounds, Wistar rats

## Abstract

Background: Cardiotoxicity remains a major concern in breast cancer management, with implications for prognosis and quality of life. Natural compounds have shown chemopreventive potential while preserving cardiac safety. This study evaluated the cardiac effects of a *Santolina chamaecyparissus* aqueous extract (SCE), characterized by high levels of 1,3-*O*-dicaffeoylquinic acid and myricetin-*O*-glucuronide, in female Wistar rats subjected to *N*-methyl-*N*-nitrosourea (MNU)-induced mammary carcinogenesis. Methods: Twenty-eight animals were assigned to four groups (*n* = 7/group): control (CTRL), MNU-induced (IND), SCE-supplemented (SCE), and MNU-induced SCE-supplemented (SCE+IND). SCE was administered in water (120 µg/mL) for 20 weeks, and MNU was injected intraperitoneally (50 mg/kg) at 50 days of age. At the end of the experiment, body and heart weights were recorded, creatine kinase MB (CK-MB) concentrations assessed, and echocardiography performed to evaluate cardiac structure and function. Results: Final body and relative heart weights did not differ among groups. CK-MB was lower in SCE-supplemented groups compared with CTRL and IND (*p* < 0.05). IND animals exhibited a hyperdynamic functional profile accompanied by impaired ventricular filling, which was attenuated in SCE+IND animals (*p* < 0.05). Cardiac structural parameters were largely preserved with SCE, despite an increased left ventricular mass (*p* < 0.05). Conclusions: SCE did not induce adverse cardiac effects and partially mitigated early carcinogenesis-associated cardiac alterations under the tested conditions, supporting its cardiac safety as a potential phenolic-rich chemopreventive strategy.

## 1. Introduction

Breast cancer remains the most frequently diagnosed malignancy among women and a leading cause of cancer-related mortality worldwide [1]. Despite significant advances in prevention and therapy, current systemic treatments often exert considerable off-target toxicity [2], particularly affecting the cardiovascular system [3]. Anthracyclines, endocrine therapies, and targeted agents are well known for inducing cardiomyocyte damage, oxidative stress, mitochondrial dysfunction, impaired ventricular performance, and long-term cardiotoxicity [4,5,6,7,8]. These adverse effects have prompted increasing interest in strategies that can simultaneously influence mammary carcinogenesis and preserve cardiovascular integrity. Importantly, cardiovascular alterations in oncology are not solely a consequence of anticancer therapies. Accumulating evidence indicates that cancer itself acts as a systemic disease capable of inducing early and frequently subclinical cardiac dysfunction [9,10,11]. Tumour development is associated with the release of tumour-derived factors, particularly pro-inflammatory cytokines such as tumour necrosis factor (TNF)-α and interleukin (IL)-6 [12], as well as increased production of reactive oxygen species (ROS) [13], which contribute to systemic inflammatory and oxidative stress responses that can adversely affect cardiac structure and function. Such subclinical alterations may predispose the myocardium to subsequent injury and contribute to long-term cardiovascular morbidity [14]. Accordingly, comprehensive cardiovascular assessment is increasingly recognized as an essential component of preclinical breast cancer research, particularly in studies evaluating candidate chemopreventive agents.

Natural compounds have emerged as promising candidates in cancer chemoprevention due to their wide availability, favourable safety profile, and multimodal biological actions [15,16]. Several plant-derived extracts have shown the capacity to modulate molecular pathways involved in carcinogenesis, including oxidative stress, inflammation, angiogenesis, proliferation, and oestrogen signalling [17,18,19]. *Santolina chamaecyparissus* L., a flowering plant species native to the western and central Mediterranean, possesses antioxidant, anti-inflammatory, antidiabetic, and anticancer activities and has recently gained attention as a potential source of bioactive compounds [20,21]. In particular, its aqueous extract is enriched in dicaffeoylquinic acids and flavones, phenolic constituents reported to exert antioxidant, anti-inflammatory, and cardioprotective actions, including attenuation of chemotherapy- and diabetes-related cardiac injury through modulation of oxidative stress, apoptosis, and adverse remodelling [22]. Given the promising chemopreventive potential and overall lack of hepatic and nephrotoxicity previously assessed with aqueous extract of *Santolina chamaecyparissus* (SCE) [23], a critical next step is to evaluate its cardiac effects using direct structural and functional measurements. Echocardiography offers a non-invasive, sensitive, and reproducible approach to investigate ventricular geometry, systolic and diastolic performance, and right-sided or pulmonary artery dynamics, while enabling detection of subclinical functional alterations and integrated assessment of ventricular–vascular coupling in preclinical models [24,25].

Given the emerging recognition of cancer-associated cardiac dysfunction and the need for comprehensive cardiovascular evaluation in long-term chemoprevention strategies, the present study aimed to characterize cardiac structure, systolic and diastolic function, and systemic and pulmonary hemodynamics in female Wistar rats subjected to *N*-methyl-*N*-nitrosourea (MNU)-induced mammary carcinogenesis, with or without SCE supplementation. By integrating biochemical and comprehensive echocardiographic assessments, this work sought to determine whether SCE induces adverse cardiovascular effects and to explore its potential to modulate early cardiac functional alterations associated with tumour development.

## 2. Materials and Methods

### 2.1. Extract Preparation and Characterization

The SCE used in this study corresponded to the same plant material batch and dry aqueous extract previously prepared and chemically characterized in Azevedo et al. [23], and no new extraction batch was produced for the present cardiac assessment. Briefly, the aerial parts of *S. chamaecyparissus*, purchased from Herbes del Molí (Alicante, Spain), were subjected to aqueous extraction by maceration. Plant material was mixed with distilled water at a solid-to-liquid ratio of 1:30 (*w*/*v*) and maintained under continuous agitation (150 rpm) at room temperature (25 °C) for 1 h. The mixture was filtered through Whatman no. 4 paper, and the residual plant material was re-extracted with an additional equivalent volume of water under identical conditions to maximize phenolic recovery. Combined filtrates were concentrated at 35 °C under reduced pressure using a rotary evaporator and subsequently frozen and lyophilized to obtain the dry extract. The extraction yield, calculated as dry extract weight relative to the initial dry plant material weight, was 8.2%.

Chemical characterization of SCE by LC-DAD-ESI/MS was performed on a Dionex Ultimate 3000 UPLC system (Thermo Scientific, San Jose, CA, USA) coupled to a Diode Array Detector (DAD, at 280, 330 and 370 nm) and an Ion Trap Linear LTQ XL mass spectrometer (Thermo Finnigan, San Jose, CA, USA) with an electrospray ionisation (ESI) source, as previously reported in Azevedo et al. [23]. Data acquisition, processing and interpretation were performed with Xcalibur software version 2.2 (ThermoFinnigan, San Jose, CA, USA) and compounds were identified by comparing retention time (RT), UV-Vis spectra, pseudomolecular ions, MS^2^ fragmentation patterns, and available commercial standards/literature data. Quantification was performed using calibration curves (R^2^ ≥ 0.999) obtained from available standards and expressed as µg/mL of extract. The extract was predominantly composed of phenolic acids, mainly 1,3-*O*-dicaffeoylquinic acid (approximately 44% of total phenolic content), and flavonoids, especially myricetin-*O*-glucuronide (approximately 10% of total phenolic content). The complete LC-DAD-ESI/MS characterization table of SCE is provided in Supplementary Table S1.

### 2.2. Experimental Design

#### 2.2.1. Ethical Considerations

All procedures were conducted in accordance with current Portuguese (Decree-Law No. 113/2013) and European legislation (Directive 2010/63/EU) on the protection of animals used for scientific purposes and the ARRIVE 2.0 guidelines for the reporting of animal research. The study protocol was reviewed and approved by the institutional Ethics Review Body (Órgão Responsável pelo Bem-Estar Animal), under reference 834-e-CITAB-2020 (approved on 18 February 2021), and by the Portuguese Ethics Committee for Animal Experimentation (Direção-Geral de Alimentação e Veterinária), under reference 04583 (approved on 28 March 2023).

#### 2.2.2. Animals

A total of 28 female Wistar rats (*Rattus norvegicus*), of four weeks of age (130.03 ± 2.78 g), were obtained from Envigo RMS Spain S.L. (Barcelona, Spain) and housed at the animal facilities of the University of Trás-os-Montes and Alto Douro under controlled conditions of temperature (23 ± 2 °C), relative humidity (50 ± 10%), and photoperiod (12 h light/12 h dark). Standard diet (4RF21, Mucedola, Milan, Italy) and water were provided ad libitum. Animals were kept in transparent polycarbonate cages with corn cob as bedding and provided environmental enrichment (cardboard boxes, paper rolls and shredded paper). The cages were changed once weekly or as needed.

After a one-week acclimation period with daily handling, animals were randomly allocated into four groups (*n* = 7 per group): (1) Control (CTRL), (2) MNU-induced (IND), (3) SCE-supplemented (SCE), and (4) SCE-supplemented and MNU-induced (SCE+IND). Randomization was performed using a computer-generated random sequence to ensure unbiased group allocation. The sample size was defined based on previous studies using similar rat models and endpoints, where comparable group sizes were sufficient to detect biologically meaningful effects, while also adhering to the principle of Reduction (3Rs). Animals in the SCE and SCE+IND groups received SCE in drinking water at a dose of 120 µg/mL throughout the experimental period (20 weeks). The experimental unit was defined as the individual animal, as treatments were applied and outcomes measured independently for each rat.

#### 2.2.3. Mammary Carcinogenesis Induction Protocol

In accordance with established guidelines for this model, animals in the IND and SCE+IND groups received an intraperitoneal injection of MNU (Fluorochem, Hadfield, UK) at 50 days of age (7 weeks old), at a dose of 50 mg/kg [26]. This carcinogen-induced model is widely used for the study of mammary tumorigenesis due to its reproducibility and similarity to human breast cancer in histopathological and molecular features [27]. The control groups received an intraperitoneal injection of 0.5 mL saline solution (NaCl 0.9%, B Braun, Melsungen, Germany).

#### 2.2.4. Animal Monitoring and Wellbeing Assessment

Body weight was recorded weekly using a precision balance (KERN^®^ PLT 6200-2A, KERN & SOHN GmbH, Balingen, Germany). Additionally, animals were monitored daily for general health status and signs of discomfort, distress, or clinical deterioration, according to previously defined humane endpoints [28]. The wellbeing assessment included general appearance, behaviour, posture, coat and grooming, response to external stimuli, hydration status, mucosal colour, respiratory pattern, and body temperature, measured using a thermometer. Each parameter was scored according to the established welfare assessment table, and animals reaching a cumulative score of 4 or higher were considered to have met the criteria for removal from the experimental protocol.

The mammary chains of all animals were palpated weekly to detect the development of mammary tumours. When present, tumours were monitored for number, anatomical location, size and potential interference with normal bodily functions, including movement, feeding, and drinking. Tumor dimensions were measured using a calliper, and the tumour surface was inspected for signs of erosion, ulceration, necrosis, bleeding, or persistent self-induced trauma, which was also considered with the remaining clinical signs to determine whether humane endpoint criteria had been reached.

#### 2.2.5. Echocardiographic Study

At the end of the experimental period, anaesthesia was induced using a combination of ketamine (75 mg/kg; Imalgene^®^ 1000, Merial S.A.S., Lyon, France) and xylazine (10 mg/kg; Rompun^®^ 2%, Bayer S.A., Kiel, Germany), administered intraperitoneally. The absence of pedal withdrawal reflexes was confirmed before proceeding. The left thoracic region was shaved using a low-noise veterinary clipper (AESCULAP^®^ GT420 Isis, Aesculap Inc., Center Valley, PA, USA), and each animal was positioned in dorsal recumbency on a heating pad to maintain its body temperature at 37.0 ± 0.5 °C. Preheated ultrasound gel was applied to the thoracic wall to ensure adequate acoustic coupling.

Transthoracic echocardiography was performed using a high-frequency ultrasound scanner (Logiq P6^®^, General Electric Healthcare, Milwaukee, WI, USA) equipped with a 4–10 MHz linear probe (Model I739, General Electric Healthcare, Milwaukee, WI, USA) suitable for small-animal imaging, following the procedures previously described [25]. Standard parasternal long-axis (PLAX), parasternal short-axis (PSAX), apical four-chamber, and apical five-chamber views were obtained, using B-mode, M-mode, Color Doppler, and Pulsed Doppler modalities. Images were recorded and subsequently analysed using the system’s proprietary software (MicroDicom 2023.01 viewer software, Sofia, Bulgaria) by two experienced operators blinded to group allocation to avoid bias. The following measurements were obtained in PLAX view: the aorta diameter (Aod), interventricular septal thickness in diastole and systole (IVSd and IVSs, respectively), left ventricle internal diameter in diastole and systole (LVIDd and LVIDs, respectively), and posterior wall thickness in diastole and systole (LVPWd and LVPWs, respectively). In PSAX view were measured: the left ventricle short-axis diameter parallel (D1) and perpendicular (D2) to the septum, heart rate (HR), pulmonary artery diameter (PA diameter), pulmonary artery velocity-time integral (PA VTI) and pulmonary artery acceleration time (PAAT). The left atria (LA), the tricuspid annular plane systolic excursion (TAPSE), the blood flow velocity during early (E-wave) and late (A-wave) filling were determined in 4-chamber apical view. The E/A ratio was subsequently calculated by dividing the E-wave velocity by the A-wave velocity. Aortic velocity-time integral (Ao VTI) and left ventricle ejection time (LVET) were recorded in the ascending aorta in the 5-chamber apical view. Cardiac output (CO), eccentricity index (EI), ejection fraction (EF), fractional shortening (FS), left ventricle mass (LV mass), left ventricular end-diastolic volume (LVEDV), left ventricular end-systolic volume (LVESV) and stroke volume (SV) were calculated using previously validated formulas [25]. All measurements were performed over at least three consecutive cardiac cycles.

#### 2.2.6. Euthanasia

Twenty weeks after MNU injection, all animals were euthanized by anaesthetic overdose (xylazine and ketamine) followed by exsanguination via cardiac puncture, as indicated by Federation of European Laboratory Animal Science Associations. Blood samples were collected into lithium-heparin tubes (FL MEDICAL, Torreglia, Italy) and centrifuged at 1500× *g* for 10 min at 4 °C (Heraeus Labofug^TM^ 400R, Thermo Fisher Scientific, Waltham, MA, USA). The resulting plasma was aliquoted and stored at −80 °C until analysis. The heart was weighed to calculate relative organ weight.

### 2.3. Plasma Biochemistry

Plasma concentrations of creatine kinase MB (CK-MB) were determined in an AutoAnalyzer (Prestige 24i, Cormay PZ, Diamond Diagnostics, Holliston, MA, USA).

### 2.4. Statistical Analysis

The statistical analysis was conducted using GraphPad Prism Version 10.5.1 (GraphPad Software, San Diego, CA, USA). The Shapiro–Wilk and Levene tests were used to determine the normality and homogeneity of variances of the data, respectively. A one-way analysis of variance (ANOVA) followed by the Tukey multiple comparisons test. In addition, because the biological interpretation of this study was centred on the modulatory effect of SCE in MNU-induced animals, only logical comparisons (CTRL vs. IND, CTRL vs. SCE, CTRL vs. SCE+IND, and IND vs. SCE+IND) were considered and highlighted. Data were expressed as mean ± standard error of the mean (SEM) for all variables. A *p*-value of less than 0.05 was considered statistically significant.

## 3. Results

Two animals from the IND group exceeded humane endpoint criteria and were excluded before the end of the experimental period (at weeks 12 and 17 of the experiment). As previously reported in Azevedo et al. [23], at the end of the study, tumour incidence was 57% in the IND group, whereas the SCE+IND group showed a lower incidence (29%) and a delay of approximately six weeks in the development of the first palpable mammary tumour.

### 3.1. Body and Heart Weights and Myocardial Injury Marker

**Figure 1:**
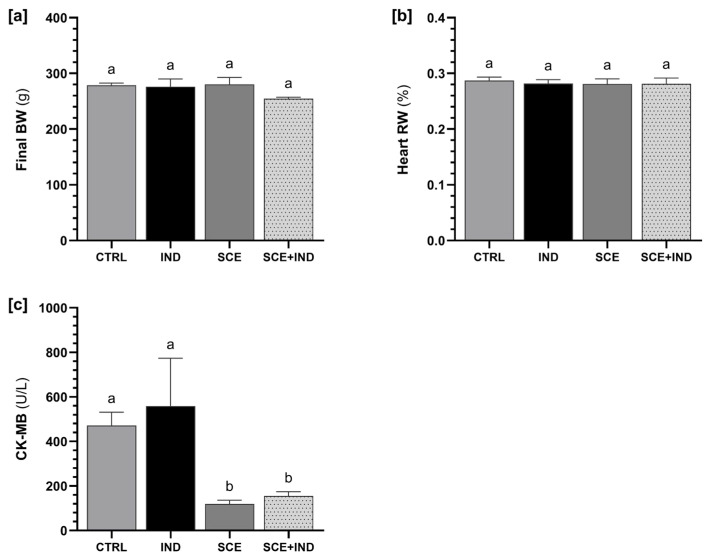
Final body weight (BW) (**a**), heart relative weight (RW) (**b**), and creatine kinase MB (CK-MB) levels (**c**) assessed at the end of the experimental period in female Wistar rats from control (CTRL), *N*-methyl-*N*-nitrosourea (MNU)-induced (IND), *S. chamaecyparissus* extract-supplemented (SCE), and SCE-supplemented MNU-induced (SCE+IND) groups. Data are presented as mean ± SEM (*n* = 7 per group, except IND where *n* = 5). Different letters denote statistically significant differences among groups (*p* < 0.05).

Body weight, relative heart weight, and CK-MB concentrations measured at the end of the experimental period are presented in Figure 1. Final body weight did not differ significantly among groups; however, a slight, non-significant reduction was observed in the SCE+IND group (*p* > 0.05). Relative heart weight was also similar among experimental groups (*p* > 0.05). In contrast, CK-MB concentrations were significantly lower in SCE and SCE+IND animals compared with CTRL and IND animals (*p* < 0.05).

### 3.2. Morphometric Parameters

**Figure 2:**
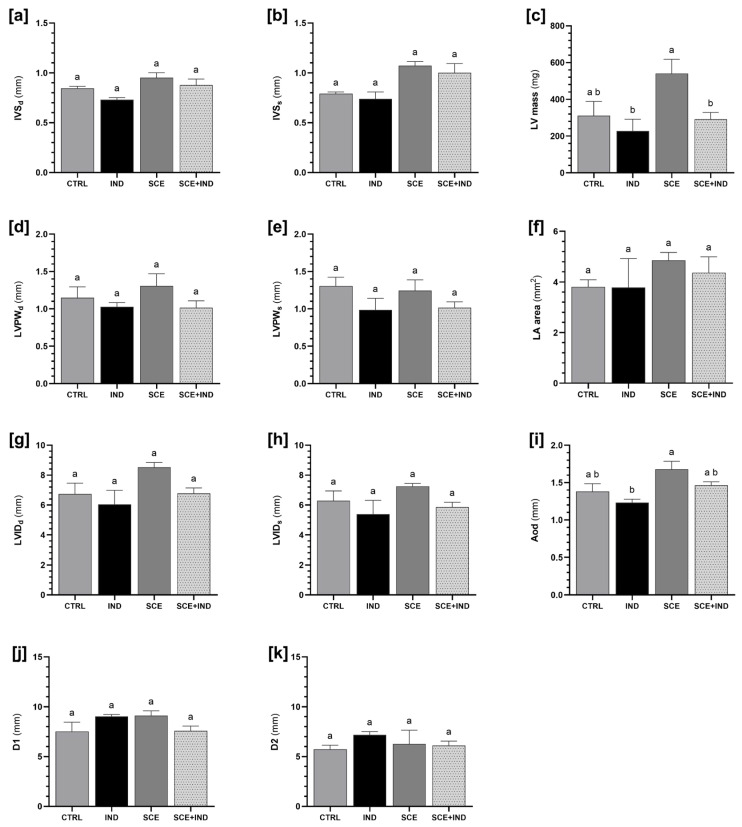
Cardiac morphological parameters assessed by transthoracic echocardiography at the end of the experimental period in female Wistar rats from control (CTRL), *N*-methyl-*N*-nitrosourea (MNU)-induced (IND), *S. chamaecyparissus* extract-supplemented (SCE), and SCE-supplemented MNU-induced (SCE+IND) groups: interventricular septal thickness in diastole (IVSd) (**a**) and systole (IVSs) (**b**), left ventricular mass (LV mass) (**c**), left ventricular posterior wall thickness in diastole (LVPWd) (**d**) and systole (LVPWs) (**e**), left atria area (LA area) (**f**), left ventricular internal diameter in diastole (LVIDd) (**g**) and systole (LVIDs) (**h**), aorta diameter (Aod) (**i**), and short-axis diameters D1 (**j**) and D2 (**k**). Data are presented as mean ± SEM (*n* = 7 per group, except IND where *n* = 5). Different letters denote statistically significant differences among groups (*p* < 0.05).

Cardiac morphometric parameters are summarized in Figure 2. IVSd and IVSs did not differ significantly among groups. LV mass, however, was significantly higher in animals supplemented with SCE compared with both MNU-induced groups (IND and SCE+IND) (*p* < 0.05). No significant differences were observed in LVPWd and LVPWs or LVIDd and LVIDs, LA area, and D1 and D2 among groups (*p* > 0.05). Aod was significantly reduced in the IND group when compared with SCE group (*p* < 0.05).

### 3.3. Functional Parameters

**Figure 3:**
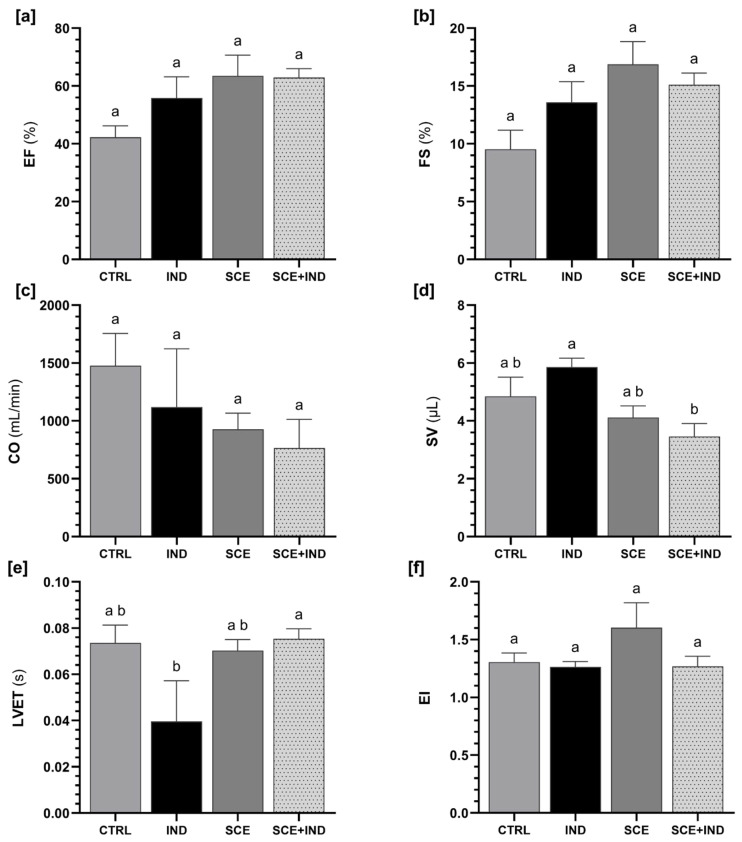
Systolic function parameters assessed by transthoracic echocardiography at the end of the experimental period in female Wistar rats from control (CTRL), *N*-methyl-*N*-nitrosourea (MNU)-induced (IND), *S. chamaecyparissus* extract-supplemented (SCE), and SCE-supplemented MNU-induced (SCE+IND) groups: ejection fraction (EF) (**a**), fractional shortening (FS) (**b**), cardiac output (CO) (**c**), stroke volume (SV) (**d**), left ventricular ejection time (LVET) (**e**), and eccentricity index (EI) (**f**). Data are presented as mean ± SEM (*n* = 7 per group, except IND where *n* = 5). Different letters denote statistically significant differences between groups (*p* < 0.05).

Systolic function parameters are presented in Figure 3. EF, FS, CO and EI did not differ significantly among experimental groups (*p* > 0.05). SV was significantly lower in the SCE+IND group when compared with IND animals (*p* < 0.05). LVET differed significantly between groups, being higher in SCE+IND compared with IND (*p* < 0.05).

**Figure 4:**
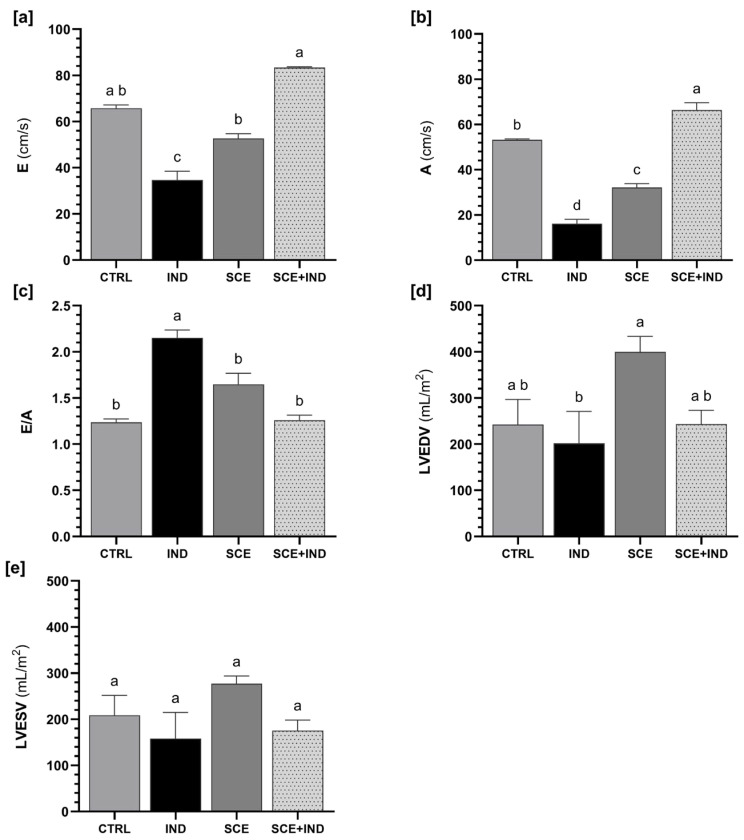
Diastolic function parameters assessed by transthoracic echocardiography at the end of the experimental period in female Wistar rats from control (CTRL), *N*-methyl-*N*-nitrosourea (MNU)-induced (IND), *S. chamaecyparissus* extract-supplemented (SCE), and SCE-supplemented MNU-induced (SCE+IND) groups: E-wave velocity (E) (**a**), A-wave velocity (A) (**b**), E/A ratio (**c**), left ventricular end-diastolic volume (LVEDV) (**d**), and left ventricular end-systolic volume (LVESV) (**e**). Data are presented as mean ± SEM (*n* = 7 per group, except IND where *n* = 5). Different letters denote statistically significant differences between groups (*p* < 0.05).

Diastolic function parameters are shown in Figure 4. Both E and A wave velocities were reduced in the IND group compared with the CTRL group (*p* < 0.05). SCE supplementation resulted in significantly higher values observed in the SCE+IND group (*p* < 0.05). Consequently, the E/A ratio was significantly elevated in IND animals compared with the remaining experimental groups (*p* < 0.05), while SCE+IND animals showed values comparable to CTRL. LVEDV was also significantly lower in the IND group, particularly when compared with the SCE group (*p* < 0.05). No significant differences were observed in LVESV among groups (*p* > 0.05).

### 3.4. Systemic and Pulmonary Hemodynamic Parameters

**Figure 5:**
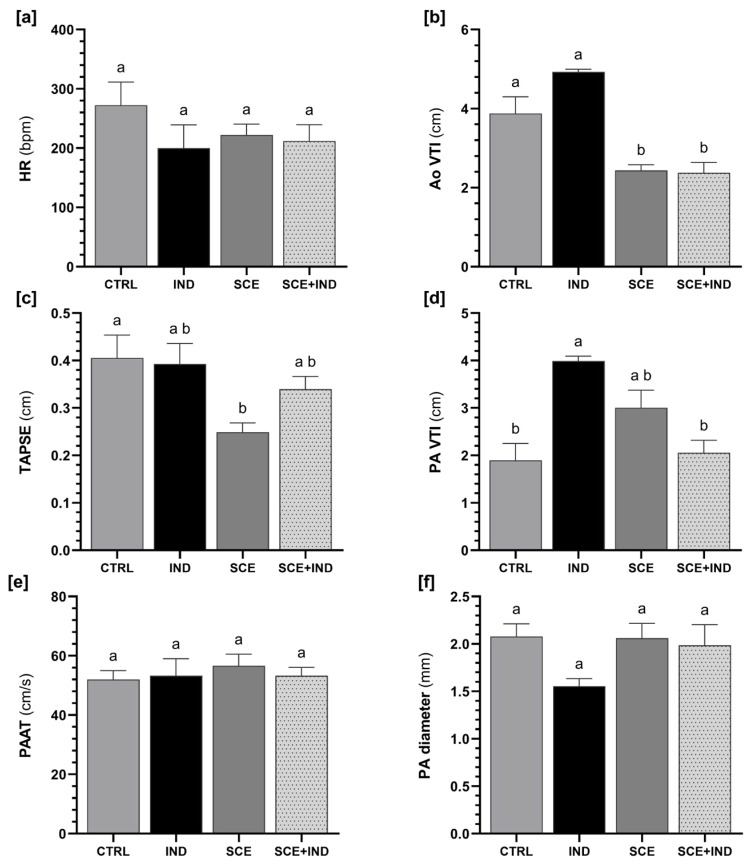
Systemic and pulmonary hemodynamic parameters assessed by transthoracic echocardiography at the end of the experimental period in female Wistar rats from control (CTRL), *N*-methyl-*N*-nitrosourea (MNU)-induced (IND), *S. chamaecyparissus* extract-supplemented (SCE), and SCE-supplemented MNU-induced (SCE+IND) groups: heart rate (HR) (**a**), aortic velocity-time integral (Ao VTI) (**b**), tricuspid annular plane systolic excursion (TAPSE) (**c**), pulmonary artery velocity-time integral (PA VTI) (**d**), pulmonary artery acceleration time (PAAT) (**e**), and pulmonary artery diameter (PA diameter) (**f**). Data are presented as mean ± SEM (*n* = 7 per group, except IND where *n* = 5). Different letters denote statistically significant differences between groups (*p* < 0.05).

Systemic and pulmonary hemodynamic parameters are summarized in Figure 5. HR did not differ significantly between experimental groups (*p* > 0.05). In contrast, Ao VTI differed significantly among treatments, with lower values observed in supplemented animals (SCE and SCE+IND groups) when compared with CTRL and IND animals (*p* < 0.05). Regarding TAPSE, only the SCE group presented lower values compared with the remaining groups (*p* < 0.05). PA VTI was significantly higher in IND animals compared with CTRL (*p* < 0.05). SCE+IND presented a significantly lower PA VTI value than IND (*p* < 0.05), being comparable to CTRL. PAAT and PA diameter did not differ significantly among groups (*p* > 0.05).

## 4. Discussion

This study provides a comprehensive evaluation of cardiac structure, systolic and diastolic function, and systemic and pulmonary hemodynamics in female Wistar rats subjected to MNU-induced mammary carcinogenesis and supplemented with an *S. chamaecyparissus* aqueous extract. Building on previous work demonstrating delayed tumour onset and reduced tumour incidence with SCE supplementation [23], the present study extends these observations by characterizing the cardiac phenotype associated with mammary carcinogenesis and assessing whether long-term exposure to SCE induces adverse or modulatory cardiovascular effects. To our knowledge, this is the first study to characterize how a phenolic-rich plant extract modulates the cardiac phenotype associated with MNU-induced mammary carcinogenesis using echocardiography.

As expected for this model, two animals in the IND group reached humane endpoints before completion, reflecting the aggressiveness of MNU-induced mammary tumour development [29]. Although the tumour results were not the primary focus of the present study, it is important to mention that tumour frequency was lower and tumour onset delayed in SCE+IND animals, consistent with previously reported chemopreventive effects of phenolic compounds [30,31]. These tumour-related differences were not accompanied by changes in final body weight or relative heart weight, suggesting that neither MNU-induced carcinogenesis nor SCE supplementation induced overt systemic effects or gross cardiac morphological alterations. CK-MB concentrations were higher in CTRL and IND groups compared to SCE-supplemented animals, suggesting a protective effect of supplementation on cardiac muscle. However, given the limited diagnostic specificity of CK-MB as a standalone marker of myocardial injury [32], these results should be interpreted cautiously and considered complementary to functional assessment by echocardiography. A substantial body of experimental evidence indicates that plant-derived phenolic compounds exert cardioprotective actions primarily through indirect mechanisms, including attenuation of oxidative stress, modulation of inflammatory signalling, and improvement of endothelial function [33,34,35], rather than inducing acute changes in cardiac morphology or contractility. In specific, caffeoylquinic acid-rich extracts and isolated isomers have been shown to attenuate myocardial injury by reducing oxidative stress, inflammatory signalling, and biochemical markers of cardiac damage in several experimental models [36,37,38].

Importantly, the concentration of SCE used in the present study (120 µg/mL in drinking water) did not induce any signs of systemic or organ toxicity, as reflected by the absence of hepatic or renal toxicity in vivo observed previously [23]. Together, these observations support the overall safety of the selected concentration for long-term administration and indicate that the observed cardiac effects are unlikely to be confounded by extract-related toxicity. From a translational perspective, this regimen corresponds to a low-dose chronic exposure to phenolic compounds for humans; although direct dose extrapolation requires formal allometric scaling, it is therefore compatible with long-term use in a chemopreventive context.

Animals with MNU-induced mammary cancer and not supplemented with SCE showed a distinct cardiac functional profile characterized by preserved global systolic performance but altered systolic timing and diastolic filling. This observation is particularly relevant given that chemically induced mammary carcinogenesis using MNU has also been employed as a model of cancer-associated cachexia [39], a condition frequently linked to cardiovascular dysfunction [40]. Despite unchanged EF, FS, and CO, animals from the IND group exhibited increased SV together with reduced LVET. This hyperdynamic systolic pattern is increasingly recognized in cancer-related cardiac phenotypes and may reflect compensatory responses to systemic inflammation [41] and altered metabolic pathways [42] associated with tumour development, even preceding possible cardiotoxic chemotherapy [43]. Importantly, this hyperdynamic profile was accompanied by significant changes in diastolic parameters, including reduced E and A wave velocities, an elevated E/A ratio, and decreased LVEDV. These findings are consistent with early diastolic dysfunction [44], which is now recognized as a sensitive marker of subclinical cardiac involvement in oncological conditions [45]. Although the magnitude of the observed functional changes was moderate, the detected alterations in diastolic filling and systolic timing are clinically meaningful, as early diastolic dysfunction and subtle changes in ventricular dynamics are recognized as sensitive indicators of subclinical cardiac involvement in oncology patients.

SCE supplementation did not induce adverse structural or functional cardiac effects in either healthy or tumour-bearing animals. Left ventricle wall thicknesses, chamber dimensions, and indices of global systolic performance remained unchanged across groups. Notably, animals receiving SCE alone exhibited an increase in LV mass without simultaneous changes in wall thickness or systolic function, which may reflect a physiological adaptation rather than pathological hypertrophy [46], although confirmation would require complementary myocardial histological or molecular analyses. The oral route of administration was selected to reflect the most relevant and realistic mode of human exposure [47]. This approach also allows continuous, low-dose intake that better mimics chronic dietary consumption of phenolic compounds and avoids pharmacokinetic artefacts associated with parenteral delivery. In tumour-bearing animals, the direct comparison between IND and SCE+IND groups showed that SCE supplementation was associated with normalization of several functional parameters altered by MNU exposure. In particular, LVET was longer in SCE+IND animals compared with IND animals, suggesting attenuation of the previously discussed hyperdynamic systolic timing observed in untreated tumour-bearing rats. More importantly, SCE supplementation partially restored diastolic filling parameters, with higher E and A wave velocities, a lower E/A ratio, and increased LVEDV compared with the IND group. These direct differences between IND and SCE+IND indicate that SCE improved ventricular filling dynamics [44] and that it may modulate early diastolic alterations associated with mammary carcinogenesis. Similarly, PA VTI was elevated in IND animals and reduced to CTRL levels in SCE+IND animals, paralleling the normalization observed in left ventricular systolic timing and supporting the concept that SCE attenuates the hyperdynamic circulatory state associated with tumour development. From a translational perspective, this functional improvement is noteworthy because breast cancer therapies, particularly anthracyclines and anti-human epidermal growth factor receptor-2 agents, are generally associated with cardiac dysfunction that is typically detected as early changes in LV function [3,48,49]. This highlights the ongoing clinical need for safer therapeutic strategies that effectively control tumour progression while minimizing cardiovascular risk, namely the use of natural compounds. Importantly, the results observed in this study regarding the normalization of functional and hemodynamic parameters are consistent with previous studies evaluating some of the major phenolic compounds in SCE in experimental models of cardiac stress. Myricetin administration restored left ventricular systolic and diastolic parameters, including LVEDV, LVESV, EF and FS, in lipopolysaccharide-induced cardiac injury and diabetic cardiomyopathy in male C57BL/6J mice [50,51]. Similarly, chlorogenic acid improved EF, FS, LVEDV, LVESV, as well as left ventricular end-diastolic and end-systolic diameters, in male C57BL/6 mice subjected to transverse aortic constriction-induced pressure overload [52]. These studies indicate that phenolic compounds act primarily as modulators of cardiac function and, in this context, the functional improvements observed with SCE supplementation are most plausibly attributed to systemic modulation of oxidative and inflammatory pathways, rather than to direct inotropic or hypertrophic effects.

Nonetheless, a few limitations should be acknowledged, namely that cardiac assessments were performed at a single terminal time point, precluding evaluation of temporal progression of functional changes. In addition, the absence of myocardial histological, molecular, or metabolic analyses limits mechanistic interpretation of the observed functional adaptations. Nevertheless, these findings provide a valuable functional framework for future studies investigating myocardial signalling pathways, oxidative stress markers, or fibrosis in the context of cancer-associated cardiac dysfunction and natural compound supplementation. Importantly, phenolic-rich extracts may exert distinct effects on cardiac function even when their phenolic profiles partially overlap, as relative abundance of phenolics and the presence of additional minor bioactive constituents can result in different cardiac responses [53,54] and must be considered when interpreting the effects of complex plant extracts.

## 5. Conclusions

In conclusion, mammary carcinogenesis induced by MNU is associated with early and predominantly diastolic cardiac functional alterations in female Wistar rats, accompanied by a hyperdynamic systolic profile. Long-term supplementation with a *S. chamaecyparissus* aqueous extract does not induce adverse cardiac remodelling and partially attenuates these functional changes to control levels, particularly at the level of ventricular filling dynamics. Together with previous evidence of chemopreventive efficacy and systemic safety, these results support the cardiovascular tolerability of SCE and highlight its potential to modulate subclinical cardiac dysfunction during cancer development. Further mechanistic studies are warranted to clarify the myocardial pathways underlying these effects and to explore the broader relevance of SCE in cardio-oncology settings. This work contributes to the growing field of cardio-oncology and supports the further exploration of phenolic-rich plant extracts as multifunctional agents capable of targeting both tumour progression and cardiovascular health.

## Data Availability

The original contributions presented in this study are included in the article/Supplementary Material. Further inquiries can be directed to the corresponding authors.

## Supplementary Materials

The following supporting information can be downloaded at https://www.mdpi.com/article/10.3390/cimb48060599/s1.

## Abbreviations

The following abbreviations are used in this manuscript:

A	A-wave velocity
Ao VTI	Aortic velocity-time integral
Aod	Aorta diameter
CK-MB	Creatine kinase MB
CO	Cardiac output
CTRL	Control group
E	E-wave velocity
EF	Ejection fraction
EI	Eccentricity index
FS	Fractional shortening
HR	Heart rate
IND	MNU-induced group
IVSd	Interventricular septal thickness in diastole
IVSs	Interventricular septal thickness in systole
LA	Left atria
LV	Left ventricular
LVEDV	Left ventricular end-diastolic volume
LVESV	Left ventricular end-systolic volume
LVET	Left ventricular ejection time
LVIDd	Left ventricular internal diameter in diastole
LVIDs	Left ventricular internal diameter in systole
LVPWd	Left ventricular posterior wall thickness in diastole
LVPWs	Left ventricular posterior wall thickness in systole
MNU	*N*-methyl-*N*-nitrosourea
PA	Pulmonary artery
PA VTI	Pulmonary artery velocity-time integral
PAAT	Pulmonary artery acceleration time
RW	Relative weight
SCE	*Santolina chamaecyparissus* extract
SV	Stroke volume
TAPSE	Tricuspid annular plane systolic excursion
