# Supplementary material for: Cardiac Modulation by Santolina chamaecyparissus Aqueous Extract in a Rat Model of Mammary Carcinogenesis
Source: Curr Issues Mol Biol. 2026 Jun 5;48(6):599. doi: 10.3390/cimb48060599 (PMC13297802; doi:10.3390/cimb48060599)
Supplement: Supplementary file 1 [file cimb-48-00599-s001.zip › cimb-4331022-supplementary.pdf]

Supplementary material

Table S1. Phenolic profile (LC-DAD-ESI/MS analysis) of the *Santolina chamaecyparissus* aqueous extract administered in the present study. Values are expressed as μg/mL of extract and presented as mean ± standard deviation. Adapted from Azevedo et al. [23], published under CC BY 4.0 license.

Peak	Rt (min)	Λmax (nm)	$[M-H]^-$ m/z	MS^2 (m/z)	Tentative identification	Quantification (μg/mL extract)
1	4.35	325	353	191(100), 179(28), 173(2), 135(9)	3-*O*-Caffeoylquinic acid[1]	7.7 ± 0.1
2	4.90	325	515	353(11), 341(6), 323(100), 191(61), 179(6), 161(18)	5-*O*-Caffeoylquinic acid hexoside[1]	6.8 ± 0.1
3	5.99	323	353	191(22), 179(52), 173(100), 135(13)	5-*O*-Caffeoylquinic acid[1]	6.81 ± 0.02
4	6.41	326	707	353(100)	dimer of 3-*O*-Caffeoylquinic acid[1]	45.1 ± 0.5
5	6.68	327	707	353(100)	dimer of 5-*O*-Caffeoylquinic acid[1]	2.85 ± 0.03
6	8.85	335	593	503(33), 473(100), 383(26), 353(53), 341(6)	Apigenin-C-hexoside-C-hexoside[2]	8.6 ± 0.1
7	14.02	352	479	317(100)	Myricetin-3-*O*-hexoside[3]	4.1 ± 0.04
8	15.08	339	463	301(100)	Quercetin-3-*O*-galactoside[3]	12.3 ± 0.2
9	15.65	338	677	515(100), 353(21)	1,3,5-*O*-tricaffeoylquinic acid[1]	4.1 ± 0.1
10	16.71	342	463	301(100)	Quercetin-3-*O*-glucoside[3]	2.17 ± 0.02
11	17.16	341	565	521(100), 479(23), 317(18)	Myricetin-*O*-malonylhexoside[3]	3.8 ± 0.04
12	17.68	350	493	331(100), 317(10)	Myricetin-*O*-glucuronide[3]	38.5 ± 0.5
13	19.14	321	515	353(100), 335(4), 253(5), 191(12), 179(2)	1,3-*O*-Dicaffeoylquinic acid[1]	165 ± 4
14	20.26	327	515	353(100), 317(5), 335(3), 299(8), 255(5), 191(2), 179(5)	1,4-*O*-Dicaffeoylquinic acid[1]	13 ± 1
15	21.58	328	515	353(100), 335(2), 191(15), 179(2), 173(2)	1,5-*O*-Dicaffeoylquinic acid[1]	27 ± 1
16	22.54	328	549	387(100)	Medioresinol-*O*-hexoside[4]	4.6 ± 0.2
17	23.17	329	515	353(100), 335(3), 299(2), 191(12), 179(5), 173(2)	4,5-*O*-Dicaffeoylquinic acid[1]	8.79 ± 0.5
18	26.26	335	725	563(100), 389(26), 341(21), 193(13), 173(8)	Apigenin-6-C-pentoside-8-c-hexoside-7-*O*-hexoside[2]	2.3 ± 0.1
19	29.64	325	457	295(100)	Coutaric acid hexoside[1]	9.4 ± 0.5
					**Total Phenolic Acids**	301 ± 7
					**Total Flavanoids**	71.7 ± 1
					**Total Phenolic Compounds**	373 ± 8

Standard curves: 1, chlorogenic acid (y = 168823x – 161172; LOD = 0.20 μg/mL; LOQ = 0.68 μg/mL); 2, apigenin-6-C-glucoside (y = 107025x + 61531; LOD = 0.10 μg/mL; LOQ = 0.53 μg/mL); 3, quercetin-3-O-glucoside (y = 34843x – 160173; LOD = 0.21 μg/mL; LOQ = 0.71 μg/mL); 4, naringenin (y = 18433x + 78903; LOD = 0.17 μg/mL; LOQ = 0.81 μg/mL).
